# Neonatal acute kidney injury and neurodevelopmental impairment: investigating associations in very low birthweight infants

**DOI:** 10.1038/s41372-025-02370-6

**Published:** 2025-07-25

**Authors:** Jeffrey E. King, Jill C. Newman, Olivia Kinsinger, Molly Mead, Megan D. Rodgers, Amy L. Ruddy-Humphries, Cassandra Coleman, David T. Selewski, Heidi J. Steflik

**Affiliations:** 1 Department of Pediatrics, Medical University of South Carolina, Charleston, SC USA; 2 Department of Pediatrics, Vanderbilt University, Nashville, TN USA

**Keywords:** Outcomes research, Kidney

## Abstract

**Objective:**

Associations between neonatal acute kidney injury (AKI) and neurodevelopmental impairment (NDI) remains understudied. We hypothesized AKI is associated with NDI in very low birthweight (VLBW) infants.

**Study design:**

A single-center, retrospective cohort study of VLBW infants with and without AKI during hospitalization evaluated in developmental clinic at 18 to 35 months of age. Associations between AKI and NDI were examined using bivariate and multivariable generalized linear mixed modeling.

**Result:**

Of 203 infants included, 64 (32%) experienced neonatal AKI and 76 (37%) developed NDI. The relative risk of NDI was significantly higher in those with AKI (RR 1.67, 95% CI 1.18–2.36; *p* < 0.01). In multivariable analysis, AKI was not an independent predictor of NDI (aRR 1.07, 95% CI 0.68–1.68; *p* = 0.77).

**Conclusion:**

In bivariate analyses, AKI was a significant predictor of NDI. In a multivariable model, AKI was no longer an independent predictor of NDI. Further investigation is warranted.

## Introduction

Neonatal acute kidney injury (AKI) has gained significant research attention over the past decade, impacting nearly 30% of all critically ill neonates [1]. AKI is a common result of several important neonatal comorbidities, including hypoxic-ischemic encephalopathy (HIE), congenital heart disease (CHD), prematurity, low birth weight (BW), and necrotizing enterocolitis (NEC) [2]. Critically ill neonates who experience AKI during hospitalization have higher mortality and increased lengths of hospitalization compared to those without AKI, emphasizing the importance of careful attention to fluid balance, recognition of risk factors, and treatment of underlying comorbidities [1–4].

Neurodevelopmental impairment (NDI) is a common complication seen in premature and low BW infants. The incidence of neurodevelopmental impairment (e.g., cerebral palsy, deafness, blindness, and intellectual disability) is roughly 45% in very low birth weight infants (VLBW; i.e., birth weight <1500 grams) and 40% in extremely preterm infants (less than 28 weeks’ gestation), highlighting the need for long term neurodevelopmental follow up [5, 6]. The association between AKI and NDI, however, remains understudied.

To bridge this knowledge gap, we investigated associations between neonatal AKI and NDI in VLBW infants. The objectives of this study were to examine the incidences of AKI and NDI, describe risk factors associated with AKI and NDI, and investigate associations between AKI and NDI in VLBW infants. We hypothesized AKI in VLBW infants is associated with NDI.

## Materials/subjects and methods

### Study population

We conducted a single-center, retrospective cohort study of VLBW infants admitted to the Medical University of South Carolina (MUSC) Neonatal Intensive Care Unit (NICU) between January 1, 2018, to December 31, 2021, and evaluated in the MUSC NICU Developmental Clinic between 18 and 35 months of age. Infants were identified using the MUSC internal Perinatal Information System (PINS) database. Exclusion criteria included: admission to MUSC NICU after 48 hours of life, congenital anomalies of the kidney and urinary tract (CAKUT), death within 48 hours of life, transition to palliative care within 48 hours of life, less than two measured serum creatinine levels, and lethal chromosomal anomalies. This protocol was approved by the MUSC Institutional Review Board with a waiver of informed consent. All findings were reported in accordance with the STROBE recommendations [7].

### Data collection

Demographic data collected included: BW, gestational age (GA), sex, race/ethnicity, reason for delivery (pre-eclampsia, chorioamnionitis, placenta accreta, placental abruption, preterm, premature rupture of membranes, intrauterine growth restriction (IUGR), placental insufficiency, preterm labor, non-reassuring fetal heart tones (NRFHT), abnormal umbilical ultrasound dopplers, maternal hemolysis, elevated liver enzymes and low platelet (HELLP) syndrome), size for gestational age (small for gestational age (SGA, <10^th^ percentile for BW), appropriate for gestational age (AGA, 10–90^th^ percentile for BW), large for gestational age ( > 90^th^ percentile for BW)), Apgar scores at birth (1, 5, and 10 minutes), abnormal head imaging during admission (intraventricular hemorrhage (IVH, Grade I–IV) or periventricular leukomalacia (PVL)), supplemental oxygen at time of discharge, length of hospitalization stay (LOS), and developmental clinic data at follow up visit (corrected age, chronologic age, weight percentile, height percentile, head circumference percentile, presence of gastrostomy tube, support services in use (early intervention, physical therapy, occupational therapy, and/or speech therapy)).

Study data were collected and managed using Research Electronic Data Capture (REDCap^®^) tools hosted by the South Carolina Clinical and Translational Science (SCTR) Institute at MUSC [8].

### Exposure: neonatal acute kidney injury

Neonatal AKI was the exposure of interest. AKI was diagnosed and staged using the modified, neonatal Kidney Disease: Improving Global Outcomes (KDIGO) serum creatinine criteria (Supplemental Fig. 1) [9]. The number of AKI events per subject (single versus recurrent) as well as AKI severity (non-severe KDIGO stage 1 versus severe KDIGO stage 2 or 3) were recorded and examined.

### Outcome: composite neurodevelopmental impairment

NDI was a composite outcome, defined as one or more of the following: hearing impairment or a developmental quotient (DQ, i.e., age-equivalent level of function divided by the current age (corrected age or chronologic age), multiplied by 100) < 75% on the Capute Scales (Cognitive Adaptive Test (CAT), Clinical Linguistic and Auditory Milestone Scale (CLAMS)), and/or Modified Peabody Developmental Motor Scale (PDMS-M) [10, 11]. Hearing impairment was assessed as any versus no impairment. If infants were evaluated multiple times between 18 and 35 months of age, data from only one developmental testing session was collected, using the testing data closest to 24 months of age. Corrected age (CA) was only used for infants ages 18 to <24 months.

Parental assessment of neurodevelopment was also evaluated, using validated tools including the Ages & Stages Questionnaires, Third Edition (ASQ®-3) and the Social-Emotional component (ASQ®-SE-2). The ASQ®-3 assesses five developmental domains (Communication, Gross Motor, Fine Motor, Problem Solving, Personal-Social). For each ASQ®-3 domain and ASQ®-SE-2, reported scores are compared to established age-specific developmental cutoffs and given a designation of ‘Pass’, ‘Borderline’, or ‘Fail’ (only ‘Pass’ or ‘Fail’ for ASQ®-SE-2). ‘Pass’ scores are ≤-1standard deviations (SD) from the mean for a given age-appropriate questionnaire, whereas ‘Borderline’ scores fall within −1 to −2 SD from the mean and ‘Fail’ scores are scores ≥-2 SD from the mean. NDI was defined by the presence of a ‘Fail’ score in any ASQ®-3 domains or on the ASQ®-SE-2.

### Statistical analysis

Descriptive statistics for the study sample were reported as frequencies and percentages for categorical data; means and SDs for normally distributed continuous data and medians and interquartile ranges (IQR: 25^th^ and 75^th^ percentiles) for non-parametric continuous data. Comparisons by AKI status (present or absent) were made using Chi-square, Fisher’s exact, Student’s t, or Wilcoxon rank sum tests, as appropriate.

Bivariate analyses to test for differences between AKI status and NDI (primary outcome) and AKI status with secondary outcomes were performed using generalized linear mixed modeling (GLMM) for binary outcomes. Relative risks (RR) were reported, as well as 95% confidence intervals (CIs) and *p*-values. AKI status was categorized as present or absent and secondary outcomes were categorized as described above.

Bivariate and multivariable GLMMs were used to examine associations between NDI, AKI, demographic and clinical characteristics. The outcomes were reported as both unadjusted and adjusted relative risks (aRR), along with their corresponding 95% CIs and *p*-values. Covariates that demonstrated a statistically significant association with NDI in a bivariate analysis were included in the multivariable model (BW, GA, pre-eclampsia, supplemental oxygen at discharge, and LOS). The multivariate model was checked for multicollinearity within the predictors. Variance Inflation Factors (VIFs) and tolerance were examined to determine if there were multicollinearity issues present between the covariates. No VIF was greater than 3.8 and no tolerance less than 0.25, indicating there were not multicollinearity issues within the model predictors.

Frequencies and percentages were reported for parental questionnaires by AKI status. Chi square tests were used to test for differences in pass, borderline, and fail questionnaire cut points with AKI status, and *p*-values were reported. Descriptive statistics for neurodevelopmental test scores, at 75% and 85% cut points, were reported as means ± SDs, or medians and IQR. Data is presented for the total cohort, as well as by AKI and NDI statuses.

A *p*-value of <0.05 was considered statistically significant. All analyses were performed using SAS software, Version 9.4 of the SAS System. Copyright © 2025 SAS Institute Inc. SAS and all other SAS Institute Inc. product or service names are registered trademarks or trademarks of SAS Institute Inc., Cary, NC, USA.

## Results

### Study population

In total, 1009 VLBW infants were admitted to the MUSC NICU from January 1, 2018, to December 31, 2021, with 783 infants meeting initial screening inclusion criteria. Out of the 783 included, 456 (58%) VLBW infants were seen at the MUSC NICU Developmental Clinic, with 203 VLBW infants evaluated between 18 and 35 months of age and included in the final analysis cohort (Supplemental Fig. 2).

### Neurodevelopmental Impairment

In our cohort, 76 (37%) experienced NDI. Key differences noted in those with NDI compared to those without, included lower BW, younger GA, less maternal pre-eclampsia, more supplemental oxygen at discharge, and longer LOS (all *p* < 0.01) (Table 1). Supplemental Table 1 details AKI staging and severity by NDI status. Supplemental Table 2 details CAT, CLAMS, and PDMS-M scores for the entire cohort as well as by NDI and AKI status.

**Table 1:** Demographic Characteristics of Infants by Neurodevelopmental Impairment Status *n* (%).

Demographic Characteristics	Overall n = 203	Neurodevelopmental Impairment (NDI)		
		Present *n* = 76 (37.4)	Absent *n* = 127 (62.6)	*p* value^b^
Birth Weight (grams)	1021 ± 277	911 ± 252	1087 ± 271	**<0.001**
Gestational Age (weeks)	27.9 ± 2.6	27.1 ± 2.8	28.4 ± 2.4	**<0.001**
Male	72 (55.4)	44 (57.9)	59 (46.5)	0.1147
Race/Ethnicity				0.1077
White/Caucasian	74 (36.6)	25 (32.9)	49 (38.9)	
Black/African American	109 (54.0)	44 (57.9)	65 (51.6)	
Hispanic	14 (6.9)	3 (4.0)	11 (8.7)	
Other	5 (2.5)	4 (5.3)	1 (0.8)	
Reason for Delivery				
Pre-eclampsia	78 (38.4)	18 (23.7)	60 (47.2)	**<0.001**
Chorioamnionitis	11 (5.4)	3 (4.0)	8 (6.3)	0.5419
Placenta Accreta	0	0	0	-
Placental Abruption	20 (9.9)	7 (9.2)	13 (10.2)	0.8124
Preterm Premature Rupture of Membranes	30 (14.8)	15 (19.7)	15 (11.8)	0.1236
IUGR	17 (8.4)	8 (10.5)	9 (7.1)	0.3919
Placental Insufficiency	0	0	0	-
Preterm Labor	71 (35.0)	35 (46.1)	36 (28.4)	**0.011**
Non-reassuring Fetal Heart Tones	37 (18.2)	14 (18.4)	23 (18.1)	0.9557
Abnormal Dopplers	12 (5.9)	7 (9.2)	5 (3.9)	0.1369
Maternal HELLP	12 (5.9)	2 (2.6)	10 (7.8)	0.2171
Other	8 (3.9)	3 (4.0)	5 (3.9)	1.0000
Size for Gestational Age				0.1009
Small	51 (25.1)	24 (31.6)	27 (21.3)	
Average	147 (72.4)	49 (64.5)	98 (77.2)	
Large	5 (2.5)	3 (4.0)	2 (1.6)	
Apgar				
1 minute	4.7 ± 2.3	4.6 ± 2.4	4.7 ± 2.2	0.8632
5 minutes	7.1 ± 1.7	6.9 ± 1.9	7.2 ± 1.6	0.2126
10 minutes (n = 61)	7.1 ± 1.3	7.0 ± 1.3	7.2 ± 1.4	0.4622
Abnormal Head Imaging during Admission^a^				
No IVH	131 (64.5)	41 (54.0)	90 (70.9)	**0.015**
Grade I/II	53 (26.1)	25 (32.9)	28 (22.1)	0.0886
Grade III/IV	18 (8.9)	9 (11.8)	9 (7.1)	0.2487
PVL	4 (2.0)	2 (2.6)	2 (1.6)	0.6311
Supplemental FiO2 at Discharge	43 (21.3)	25 (33.3)	18 (14.2)	**<0.002**
Length of Hospitalization (days)	79 [50.5, 110.5]	93 [68, 119.5]	67.5 [43, 100]	**<0.001**
Developmental Clinic Follow Up Data				
Adjusted Age at Visit (months)	25.0 ± 4.4	25.7 ± 4.6	24.6 ± 4.3	0.0837
Chronologic Age at Visit (months)	25.9 ± 3.5	26.5 ± 3.7	25.5 ± 3.3	**0.045**
Weight at Visit (percentile)	21 [5,53]	14 [5,43]	29 [8,62]	**0.014**
Height at Visit (percentile)	24 [9,53]	18.5 [4,41]	27 [11,58]	**0.035**
Head Circumference at Visit (percentile)	31 [11,67]	19 [6.5, 67.5]	34 [13,67]	**<0.050**
Gastrostomy Tube Present at Visit	14 (6.9)	11 (14.5)	3 (2.4)	**0.003**
Support Services in use at Visit^۱^				
BabyNet (Early Intervention)	128 (63.1)	58 (76.3)	70 (55.1)	**0.003**
Physical Therapy	67 (33.0)	40 (52.6)	27 (21.3)	**<.001**
Occupational Therapy	94 (46.3)	46 (60.5)	48 (37.8)	**0.002**
Speech Therapy	98 (48.3)	51 (67.1)	47 (37.0)	**<.001**
None	54 (26.6)	8 (10.5)	46 (36.2)	**<.001**
Insurance				0.8252
Medicaid	155 (76.4)	61 (80.3)	94 (74.0)	
Private	40 (19.7)	13 (17.1)	27 (21.3)	
Tricare	5 (2.5)	2 (2.6)	3 (2.4)	
Self-Pay	1 (0.5)	0	1 (0.8)	
None	2 (1.0)	0	2 (1.6)	

Categorical data presented as count (proportion of column total). Continuous data presented as mean ± standard deviation or median [Q1,Q3].

*IUGR* intrauterine growth restriction, *HELLP* hemolysis, elevated liver enzymes, low platelets, *IVH* intraventricular hemorrhage, *PVL* periventricular leukomalacia, *Fi02* fractional inspired oxygen, AKI, acute kidney injury.

^a^Infants can have both IVH and PVL; ^۱^Multiple services may be used per infant.

^b^*p* values from Chi Square or Fisher’s Exact Test, Student’s T test or Wilcoxon Rank Sum Test.

### Acute kidney injury

Of 203 infants included, 64 (32%) experienced neonatal AKI. Major differences noted in those with AKI, compared to those without, included lower BW, younger GA, less maternal pre-eclampsia, more supplemental oxygen at discharge, and increased LOS (all *p* < 0.02). Supplemental Table 3 depicts these and other demographic characteristics and key outcomes by AKI status. Of the 64 neonates who experienced AKI, 24 (38%) developed recurrent AKI, totaling 104 episodes.

### Associations between acute kidney injury and neurodevelopmental impairment

The primary outcome, composite NDI, was examined using bivariate and multivariable analyses. In bivariate analysis, the relative risk of NDI was significantly higher in those with AKI than those without (RR 1.67, 95% CI 1.18–2.36; *p* < 0.01; Table 2). Additionally, we noted an increased relative risk of NDI among those with KDIGO Stage 2 AKI compared to KDIGO Stage 1 AKI (RR 1.73, 95% CI 1.12–2.67; *p* = 0.01, Supplemental Table 1). We did not detect significant associations when examining non-severe versus severe AKI and risk of NDI nor recurrent AKI and risk of NDI (Supplemental Table 1).

**Table 2:** Associations between acute kidney injury and neurodevelopmental impairment.

Primary outcome: composite neurodevelopmental impairment (NDI)						
	*n*	NDI		RR	95% CI	*p* value^b^
**AKI Status**	**203**	Present *n *= 76 (37.4)	Absent *n *= 127 (62.6)			
Present	64	33 (43.4)	31 (24.4)	1.67	1.18, 2.36	**0.004**
Absent	139	43 (56.7)	96 (75.6)	*ref*	–	–
**Secondary Outcomes: Individual Components of NDI Composite**						
**AKI Status**	**201**	CAT DQ^a^ < 75% *n *= 40 (19.9)	CAT DQ^a^ ≥ 75% *n *= 161 (80.1)	**RR**	**95% CI**	***p-*value**
Present	62	15 (37.5)	47 (28.2)	1.35	0.76, 2.38	0.306
Absent	139	25 (62.5)	114 (70.8)	*ref*	–	–
**AKI Status**	**203**	CLAMS DQ^a^ < 75% *n *= 56 (27.6)	CLAMS DQ^a^ ≥ 75% *n *= 147 (72.4)			
Present	64	25 (44.6)	39 (26.5)	1.75	1.13, 2.72	**<0.025**
Absent	139	31 (55.4)	108 (73.5)	*ref*	–	–
**AKI Status**	**195**	PDMS-M DQ^a^ < 75% *n *= 23 (11.8)	PDMS-M DQ^a^ ≥ 75% *n *= 172 (88.2)			
Present	61	12 (52.2)	49 (28.5)	2.40	1.12, 5.15	**0.025**
Absent	134	11 (47.8)	123 (71.5)	*ref*	–	–
**AKI Status**	**144**	Hearing Impairment *n *= 9 (6.2)	No Hearing Impairment *n *= 135 (93.8)			
Present	43	5 (55.6)	38 (28.2)	2.94	0.82, 10.52	0.098
Absent	101	4 (44.4)	97 (71.8)	*ref*	–	–

*RR* relative risk, *CI* confidence interval, *ref* reference category.

^a^Adjusted age used for DQ calculation if 18 to <24 months of age at time of evaluation; Categorical data represented as *n* (%).

^b^*p* values from generalized linear mixed models.

When examining associations between AKI and each component of the NDI composite, we detected an increased risk of CLAMS and PDMS-M DQ < 75% in those with AKI (CLAMS: RR 1.75, 95% CI 1.13–2.72; PDMS-M: RR 2.40, 95% CI 1.12–5.15; both *p* ≤ 0.03; Table 2). No significant difference was detected when examining associations between AKI and remaining NDI composite components (CAT nor hearing impairment).

In multivariable analysis, AKI was not an independent predictor of NDI (Table 3). Notably, after inclusion in the multivariate model, BW, GA, and maternal pre-eclampsia remained significant predictors of NDI (all *p* < 0.03, Table 3).

**Table 3:** Bivariate and multivariable associations between acute kidney injury and neurodevelopmental impairment.

Characteristic	Bivariate Analysis			Multivariate Analysis		
	RR	95% CI	p-value^a^	aRR	95% CI	*p* value^a^
Any AKI Episode	1.67	(1.18, 2.36)	**0.004**	1.07	(0.68, 1.68)	0.767
Birth Weight (grams)	0.99	(0.9987, 0.9993)	**<0.001**	0.998	(0.99, 1.00)	**<0.001**
Gestational Age (weeks)	0.88	(0.82, 0.94)	**<0.001**	1.17	(1.03, 1.33)	**0.020**
Pre-eclampsia	0.50	(0.32, 0.78)	**<0.003**	0.51	(0.33, 0.79)	**0.003**
Supplemental FiO2 at Discharge	1.85	(1.31, 2.61)	**0.001**	1.40	(0.95, 2.06)	0.089
Length of Hospitalization	1.01	(1.01, 1.02)	**<0.001**	1.01	(0.99, 1.01)	0.072

*RR* relative risk, *CI* confidence interval, *aRR* adjusted relative risk.

^a^*p* values from generalized linear mixed models.

**Fig. 1:**
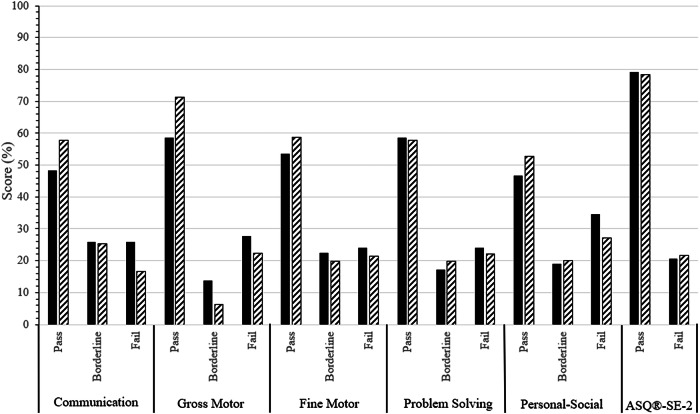
Parental assessment of neurodevelopment by developmental domain. .

When evaluating associations between AKI and parental assessments of neurodevelopment, no significant difference was noted in any ASQ®-3 domain or on the ASQ®-SE-2 (all *p* ≥ 0.5, Fig. 1).

## Discussion

AKI is common in the NICU, with frequencies as high as 48% in neonates born at 22-29 weeks’ gestation, highlighting the importance of continued research to identify both short- and long-term sequelae in this extremely vulnerable population [1, 2, 4]. In our cohort, we aimed to investigate associations between AKI and subsequent NDI, assessed by both the medical team and parents, in VLBW infants at 18 to 35 months of age. The prevalence of AKI and NDI in our cohort is comparable to rates previously reported in literature [6, 12]. Although bivariate analysis found a statistically significant association between AKI and NDI, after controlling for potential confounders, AKI was not found to be an independent predictor of NDI.

As mentioned, there is little information surrounding the relationship between neonatal AKI and NDI. Intraventricular hemorrhage (IVH), a known comorbidity seen among preterm and low birth weight infants, negatively impacts neurodevelopment [13]. Stoops et al. [14] revealed an association between AKI and IVH, demonstrating infants who had AKI during hospitalization were 1.6 times more likely to develop any grade IVH than those without AKI [14]. In addition, Sarkar et al. [15] demonstrated neonatal AKI is independently associated with hypoxic-ischemic brain lesions seen on magnetic resonance imaging (MRI) in asphyxiated newborns who underwent therapeutic hypothermia, thus establishing links between these complex organ systems [15]. Chen et al. [16] investigated the associations between oliguric and non-oliguric AKI with head circumference at term-equivalent age and neurodevelopment in 154 preterm neonates in Taiwan and found that preterm infants with oliguric AKI were at increased risk of NDI compared to those without AKI (*p* < 0.001) [16]. Distinguishing oliguric versus non-oliguric AKI in the neonatal population can be quite challenging. This is largely due to difficulties in accurately documenting urine output, as most output is recorded as mixed urine and stool, and catheters are used infrequently. In our cohort, AKI was diagnosed solely by using the serum creatinine criteria. If urine output was considered in our study for the diagnosis of AKI, there is a potential for increased AKI diagnoses within our population, based on findings in previous studies that suggest failing to utilize urine output in diagnosing AKI leads to missed cases of AKI [1]. Had this been utilized in our study, and more neonates were diagnosed with AKI, we hypothesize an association between AKI and NDI would be detected and thus support previously cited literature.

Though the gold standard testing for neurodevelopment is the Bayley Scales of Infant and Toddler Development (BSID), in this study we utilized Capute Scales (CAT and CLAMS) and PDMS-M to assess neurodevelopment at 18 to 35 months. Unfortunately, test administration time for BSID typically ranges from 30-90 minutes and requires a trained provider to administer, which makes it less accessible in many centers [17, 18]. Alternatively, the Capute Scales and PDMS-M are widely used developmental assessment tools that can be administered quickly (10-15 minutes) and in limited resource settings, making it an ideal form of developmental testing in any outpatient setting [10, 11]. Kube et al. [19] found CAT/CLAMS highly correlated with BSID, with a sensitivity of 81% and specificity of 85% in detecting overall NDI, and an even greater sensitivity and specificity when detecting severe NDI (sensitivity 100%, specificity 96%) [19].

The timing of developmental testing is an important consideration. In infants, developmental testing is limited, and thus it can be difficult to detect subtle signs of developmental delay. However, developmental testing in toddlers is extensive, placing greater developmental expectations on the toddler and thus making it easier to detect deficiencies in development. For example, language assessment at 12 months of age evaluates the infant’s ability to speak at least 2 words, whereas at 21 months of age, the toddler is expected to speak at least 20 words and, when prompted, point to at least 2 figures on a sheet of paper [10]. Garfinkle et al. (2024) demonstrated that, in infants born at <29 weeks’ gestation, the detection of developmental delay was highest when tested at 21-24 months’ CA when compared to earlier developmental testing (18-20 months’ CA) [20]. In our cohort, infants were tested between 18- and 35-months’ CA. The wide age range was due to limited patient data closest to 24 months of age. This was likely in part due to the SARS-CoV-2 pandemic, as the MUSC NICU Developmental Clinic was closed for five months (April 2020 to September 2020) and only performing virtual visits during that time. Other external socioeconomic factors, including transportation, likely played a role in follow up. Although we did not detect associations in our multivariable analysis, we suspect the limitations of testing may play a role. As such, expansion of this patient population, in addition to more longitudinal follow up, could prove to be beneficial in further characterizing associations between AKI and NDI.

We also aimed to investigate the associations between AKI and parental assessments of neurodevelopment, utilizing the ASQ®-3 and ASQ®-SE-2. The ASQ®-3 and ASQ®-SE-2 are widely accepted, validated assessment tools that have been used in several large, multicenter studies, including the Milking in Nonvigorous Infants (MINVI) randomized clinical trial [21]. This study’s approach of including both medical and parental assessments of neurodevelopment to examine NDI provides a comprehensive assessment of development and corroborated the findings by the medical team, as no association were detected in those with AKI compared to those without.

Interestingly, maternal pre-eclampsia was significantly less common in neonates with AKI and was associated with decreased risk of subsequent NDI. This observed effect of maternal pre-eclampsia among neonates with and without AKI has been demonstrated in other multi-center studies, including the AWAKEN study [1]. We hypothesize exposure to maternal magnesium prior to delivery, a common neuroprotective strategy employed by obstetricians who anticipate premature delivery and frequently provided to mothers with pre-eclampsia, positively impacts neonatal renal and central nervous system development and function [22]. Several studies have demonstrated magnesium has anti-inflammatory properties in rat models, and when hypomagnesemia is present, there is an induction of an acute-phase inflammatory response with reactive oxygen species (ROS) production [23, 24]. In neonatal disease processes with high systemic inflammatory responses (e.g., NEC, HIE), magnesium may serve as an anti-inflammatory agent that protects the kidneys from ongoing damage. Adult studies have revealed that magnesium supplementation in the intensive care unit (ICU) decreased the incidence of AKI, and hypomagnesemia is an independent risk factor for non-recovery of renal function after AKI [25, 26]. However, no studies to date have explored this relationship in neonates, and thus further investigations are underway.

As mentioned previously, this was a retrospective cohort design which inherently comes with limitations. The timing of serum creatinine measurements was performed at provider discretion without a standardized protocol, thus potentially missing AKI diagnoses. AKI was unable to be diagnosed using the modified neonatal KDIGO urine output criteria, given most neonatal output is measured as mixed urine and stool, which likely impacted the diagnosis rate of AKI within our unit. Out of the 783 included in this cohort study, only 26% (203) of patients were evaluated at follow up between 18 and 35 months of age, which was likely impacted by the SARS-CoV-2 pandemic. Lastly, though our analysis adjusted for potential confounders (e.g., BW, GA) associated with NDI and AKI, additional ones may exist that were not examined in this cohort.

## Conclusion

AKI was found to be a significant predictor of NDI in bivariate analyses; however, AKI was not found to be an independent predictor of NDI in our multivariate modeling when controlling for other specific clinical characteristics. Additional investigation is warranted to further delineate this relationship.

## Supplementary information

Supplemental Figure 1. Modified, Neonatal Kidney Disease: Improving Global Outcomes (KDIGO) Serum Creatinine Criteria

Supplemental Figure 2. Study Enrollment Flow Diagram

Supplemental Tables

## Data Availability

The datasets generated during and/or analyzed during the current study are not publicly available due IRB limitations but are available from the corresponding author on reasonable request

## References

[1] Jetton JG, Boohaker LJ, Sethi SK, Wazir S, Rohatgi S, Soranno DE, et al. Incidence and outcomes of neonatal acute kidney injury (AWAKEN): a multicentre, multinational, observational cohort study. Lancet Child Adolesc Health. 2017;1:184–94. 29732396 10.1016/S2352-4642(17)30069-X PMC5933049

[2] Coleman C, Tambay Perez A, Selewski DT, Steflik HJ. Neonatal acute kidney injury. Front Pediatr. 2022;10:842544. 35463895 10.3389/fped.2022.842544 PMC9021424

[3] Chaturvedi S, Ng KH, Mammen C. The path to chronic kidney disease following acute kidney injury: a neonatal perspective. Pediatr Nephrol. 2017;32:227–41. 26809804 10.1007/s00467-015-3298-9

[4] Starr MC, Charlton JR, Guillet R, Reidy K, Tipple TE, Jetton JG, et al. Advances in neonatal acute kidney injury. Pediatrics. 2021;148:1–9. 10.1542/peds.2021-051220 34599008

[5] Juul SE, Comstock BA, Wadhawan R, Mayock DE, Courtney SE, Robinson T, et al. A randomized trial of erythropoietin for neuroprotection in preterm infants. N Engl J Med. 2020;382:233–43. 31940698 10.1056/NEJMoa1907423 PMC7060076

[6] Molad M, Gover A, Marai Z, Lavie-Nevo K, Kessel I, Shemer-Meiri L, et al. Neurodevelopmental outcome of very low birth weight infants in the northern district of Israel: A cross-sectional study. Children (Basel). 2023;10:1–9. 10.3390/children10081320 PMC10453082 37628319

[7] von Elm E, Altman DG, Egger M, Pocock SJ, Gøtzsche PC, Vandenbroucke JP. The strengthening the reporting of observational studies in epidemiology (STROBE) statement: guidelines for reporting observational studies. J Clin Epidemiol. 2008;61:344–9. 18313558 10.1016/j.jclinepi.2007.11.008

[8] Harris PA, Taylor R, Thielke R, Payne J, Gonzalez N, Conde JG. Research electronic data capture (REDCap)-a metadata-driven methodology and workflow process for providing translational research informatics support. J Biomed Inform. 2009;42:377–81. 18929686 10.1016/j.jbi.2008.08.010 PMC2700030

[9] Zappitelli M, Ambalavanan N, Askenazi DJ, Moxey-Mims MM, Kimmel PL, Star RA, et al. Developing a neonatal acute kidney injury research definition: a report from the NIDDK neonatal AKI workshop. Pediatr Res. 2017;82:569–73. 28604760 10.1038/pr.2017.136 PMC9673450

[10] Accardo, PCA The Capute Scales: Cognitive Adaptive Test/Clinical Linguistic and Auditory Milestone Scale. Brooks Publishing: Pacific Groove, CA, USA, 2005.

[11] Watling R Peabody Developmental Motor Scales (PDMS). In: Volkmar FR, editor. Encyclopedia of Autism Spectrum Disorders. New York, NY: Springer New York; 2013;2138–40.

[12] Carmody JB, Swanson JR, Rhone ET, Charlton JR. Recognition and reporting of AKI in very low birth weight infants. Clin J Am Soc Nephrol. 2014;9:2036–43. 25280497 10.2215/CJN.05190514 PMC4255405

[13] Zhou M, Wang S, Zhang T, Duan S, Wang H. Neurodevelopmental outcomes in preterm or low birth weight infants with germinal matrix-intraventricular hemorrhage: a meta-analysis. Pediatr Res. 2024;95:625–33. 37935882 10.1038/s41390-023-02877-8 PMC10899112

[14] Stoops C, Boohaker L, Sims B, Griffin R, Selewski D, Askenazi D. The association of intraventricular hemorrhage and acute kidney injury in premature infants from the assessment of the worldwide acute kidney injury epidemiology in neonates (AWAKEN) study. Neonatology. 2019;116:321–30. 31461717 10.1159/000501708 PMC6881521

[15] Sarkar S, Askenazi DJ, Jordan BK, Bhagat I, Bapuraj JR, Dechert RE, et al. Relationship between acute kidney injury and brain MRI findings in asphyxiated newborns after therapeutic hypothermia. Pediatr Res. 2014;75:431–5. 24296799 10.1038/pr.2013.230

[16] Chen CC, Chu CH, Lin YC, Huang CC. Neurodevelopment after neonatal acute kidney injury in very preterm-birth children. Kidney Int Rep. 2023;8:1784–91. 37705902 10.1016/j.ekir.2023.06.020 PMC10496073

[17] Del Rosario C, Slevin M, Molloy EJ, Quigley J, Nixon E. How to use the bayley scales of infant and toddler development. Arch Dis Child Educ Pr Ed. 2021;106:108–12. 10.1136/archdischild-2020-319063 32859738

[18] Balasundaram P, Avulakunta ID Bayley Scales Of Infant and Toddler Development. StatPearls Publishing. 2025. 33620792

[19] Kube DA, Wilson WM, Petersen MC, Palmer FB. CAT/CLAMS: its use in detecting early childhood cognitive impairment. Pediatr Neurol. 2000;23:208–15. 11033282 10.1016/s0887-8994(00)00191-0

[20] Garfinkle J, Khairy M, Simard MN, Wong J, Shah PS, Luu TM, et al. Corrected Age at Bayley Assessment and Developmental Delay in Extreme Preterms. Pediatrics. 2024;153:1–8. 10.1542/peds.2023-063654 38186292

[21] Katheria AC, El Ghormli L, Clark E, Yoder B, Schmölzer GM, Law BHY, et al. Two-year outcomes of umbilical cord milking in nonvigorous infants: a secondary analysis of the MINVI randomized clinical trial. JAMA Netw Open. 2024;7:e2416870. 38949814 10.1001/jamanetworkopen.2024.16870 PMC11217871

[22] Rouse DJ, Hirtz DG, Thom E, Varner MW, Spong CY, Mercer BM, et al. A randomized, controlled trial of magnesium sulfate for the prevention of cerebral palsy. N Engl J Med. 2008;359:895–905. 18753646 10.1056/NEJMoa0801187 PMC2803083

[23] Bussière FI, Gueux E, Rock E, Girardeau JP, Tridon A, Mazur A, et al. Increased phagocytosis and production of reactive oxygen species by neutrophils during magnesium deficiency in rats and inhibition by high magnesium concentration. Br J Nutr. 2002;87:107–13. 11895162 10.1079/BJN2001498

[24] Regan RF, Guo Y. Magnesium deprivation decreases cellular reduced glutathione and causes oxidative neuronal death in murine cortical cultures. Brain Res. 2001;890:177–83. 11164781 10.1016/s0006-8993(00)03156-5

[25] Alves SC, Tomasi CD, Constantino L, Giombelli V, Candal R, Bristot MdeL, et al. Hypomagnesemia as a risk factor for the non-recovery of the renal function in critically ill patients with acute kidney injury. Nephrol Dial Transplant. 2013;28:910–6. 22764195 10.1093/ndt/gfs268

[26] Barbosa EB, Tomasi CD, de Castro Damasio D, Vinhas M, Lichtenfels B, de Luca Francisco V, et al. Effects of magnesium supplementation on the incidence of acute kidney injury in critically ill patients presenting with hypomagnesemia. Intensive Care Med. 2016;42:1084–5. 26928037 10.1007/s00134-016-4276-9

