# Supplementary figures and images for: Neonatal acute kidney injury and neurodevelopmental impairment: investigating associations in very low birthweight infants
Source: J Perinatol. 2025 Jul 25;45(10):1462–8. doi: 10.1038/s41372-025-02370-6 (PMC12479349; doi:10.1038/s41372-025-02370-6)

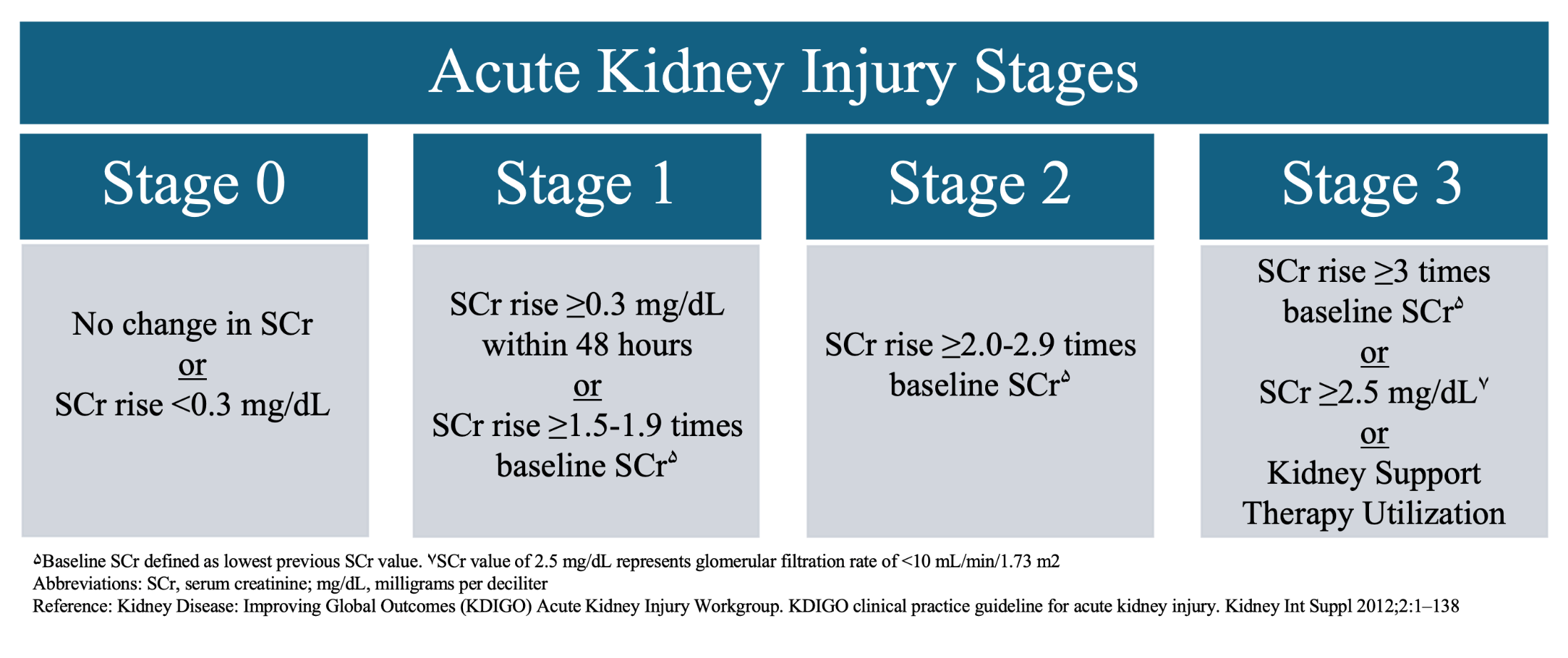

Supplement: Supplementary file 1 — Supplemental Figure 1. Modified, Neonatal Kidney Disease: Improving Global Outcomes (KDIGO) Serum Creatinine Criteria [file 41372_2025_2370_MOESM1_ESM.docx]
