# Supplementary material for: Neonatal acute kidney injury and neurodevelopmental impairment: investigating associations in very low birthweight infants
Source: J Perinatol. 2025 Jul 25;45(10):1462–8. doi: 10.1038/s41372-025-02370-6 (PMC12479349; doi:10.1038/s41372-025-02370-6)
Supplement: Supplementary file 2 — Supplemental Figure 2. Study Enrollment Flow Diagram [file 41372_2025_2370_MOESM2_ESM.docx]

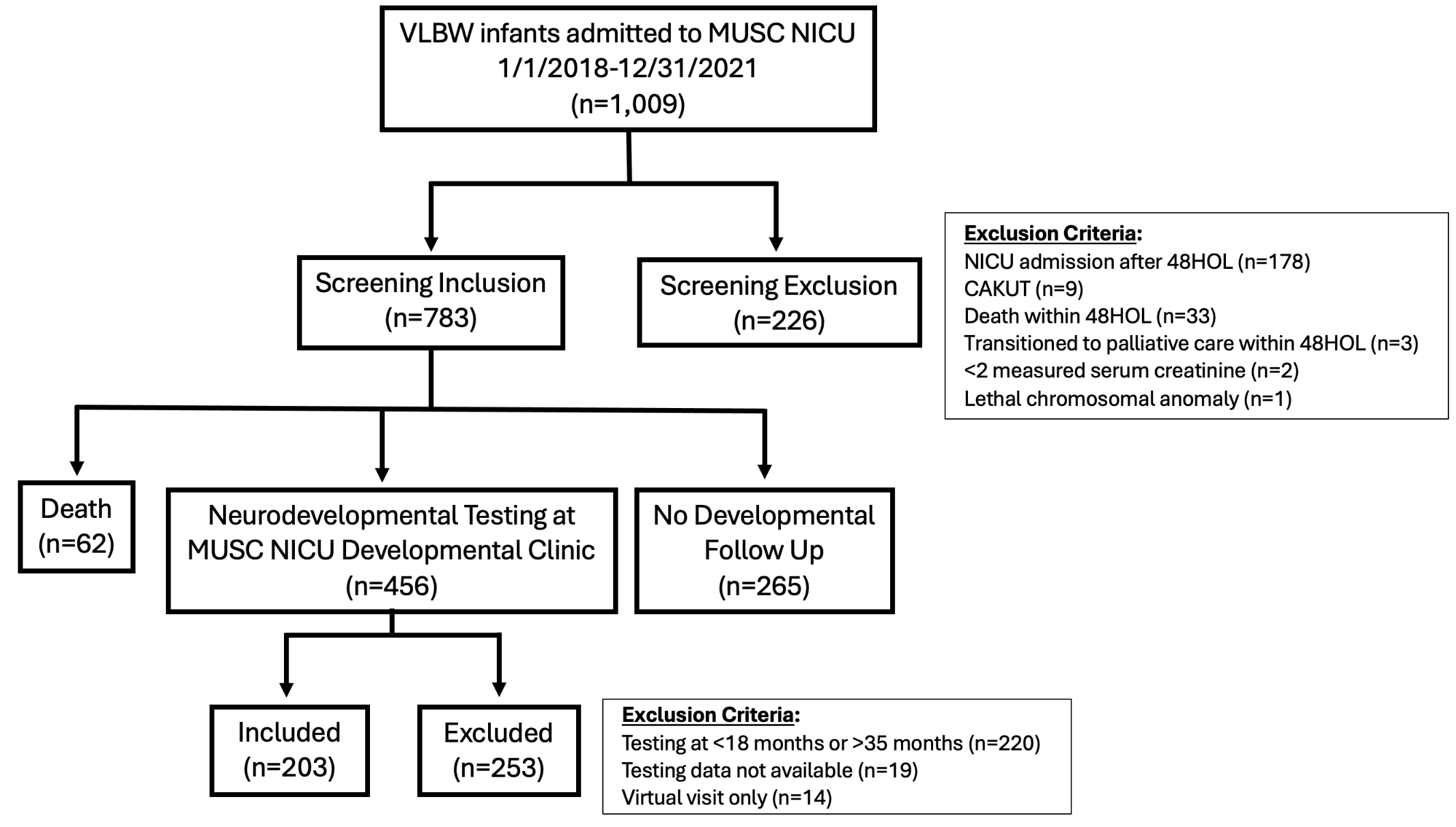


Abbreviations: VLBW, very low birthweight; MUSC, Medical University of South Carolina; NICU, neonatal intensive care unit; CAKUT, congenital anomalies of the kidney and urinary tract; HOL, hours of life
