# Supplementary material for: Neonatal acute kidney injury and neurodevelopmental impairment: investigating associations in very low birthweight infants
Source: J Perinatol. 2025 Jul 25;45(10):1462–8. doi: 10.1038/s41372-025-02370-6 (PMC12479349; doi:10.1038/s41372-025-02370-6)
Supplement: Supplementary file 3 — Supplemental Tables [file 41372_2025_2370_MOESM3_ESM.docx]

Supplemental Table 1. Presence of and risk for Neurodevelopmental Impairment by Acute Kidney Injury Stage and Severity

| **AKI Stage** | **AKI**  **Overall** | **Neurodevelopmental Impairment (NDI)** | | **RR** | **95% CI** | ***p-*value**^ح^ |
| --- | --- | --- | --- | --- | --- | --- |
|  |  | Present  n=76 (37.4) | Absent  n=127 (62.6) |  |  |  |
| **AKI Episode (n=64)** | | | | | | |
| Any AKI | 64 (31.5) | 33 (43.4) | 31 (24.4) | 1.67 | (1.18, 2.36) | **0.004** |
| No AKI | 139 (68.5) | 43 (56.6) | 96 (75.6) | *ref* |  |  |
| **AKI Stage (n=104)** | | | | | | |
| KDIGO Stage 1 | 80 (76.9) | 38 (71.7) | 42 (82.4) | *ref* |  |  |
| KDIGO Stage 2 | 21 (20.2) | 12 (22.6) | 9 (17.6) | 1.73 | (1.12, 2.67) | **0.013** |
| KDIGO Stage 3 | 3 (2.9) | 3 (5.7) | 0 | 0.83 | (0.54, 1.29) | 0.409 |
| **AKI Severity (n=104)** | | | | | | |
| Non-severe AKI^۵^ | 80 (76.9) | 38 (71.7) | 42 (82.4) | *ref* |  |  |
| Severe AKI^۷^ | 24 (23.1) | 15 (28.3) | 9 (17.6) | 1.32 | (0.89, 1.94) | 0.167 |
| **Recurrent AKI**^۹^ **(n=24)** | | | | | | |
| Recurrent AKI | 24 (37.5) | 10 (30.3) | 14 (45.2) | 0.72 | (0.42, 1.26) | 0.250 |
| Non-recurrent AKI | 40 (62.5) | 23 (69.7) | 17 (54.8) | *ref* |  |  |

Overall, 64 subjects developed acute kidney injury (AKI). AKI stage and severity (n=104) includes all AKI episodes, as some patients had multiple episodes during admission. If more than one episode of AKI during admission, severity based on most severe AKI episode

^۵^Non-severe AKI defined as Kidney Disease: Improving Global Outcomes (KDIGO) stage 1 AKI; ^۷^Severe AKI defined as KDIGO stage 2 or 3 AKI; ^۹^Multiple episodes of AKI during admission

Abbreviations: AKI, acute kidney injury; RR, relative risk; CI, confidence interval; *ref*, reference category

^ح^*p*-values from generalized linear mixed models

Supplemental Table 2. Developmental Test Scores by Acute Kidney Injury Status and Neurodevelopmental Impairment Status

| **Neurodevelopmental Test** | **Total Cohort** | | **Any AKI**  n=64 (31.5) | | **No AKI**  n=139 (69.5) | | **NDI Present**  n=76 (37.4) | | **NDI Absent**  n=127 (62.6) | |
| --- | --- | --- | --- | --- | --- | --- | --- | --- | --- | --- |
|  | n | Score | n | Score | n | Score | n | Score | n | Score |
| **CAT DQ**^۵^ | 201 | 88.1 ± 18.4 | 62 | 85.0 ± 21.0 | 139 | 89.5 ± 17.0 | 76 | 74.3 ± 18.3 | 125 | 96.6 ± 12.4 |
| DQ <85 | 70 | 68.8 ± 15.4 | 22 | 63.4 ± 19.5 | 48 | 71.2 ± 12.6 | 52 | 65.6 ± 15.1 | 18 | 77.9 ± 12.5 |
| DQ <75 | 40 | 67 [54, 71] | 15 | 55.1 ± 18.3 | 25 | 63.2 ± 12.8 | 39 | 61.0 ± 14.7 | 1 | 28.5 ± N/A |
| **CLAMS DQ**^۵^ | 203 | 84.4 ± 19.1 | 64 | 81.4 ± 20.5 | 139 | 85.7 ± 18.3 | 76 | 67.4 ± 16.5 | 127 | 94.5 ± 12.0 |
| DQ <85 | 90 | 67.7 ± 13.5 | 34 | 65.9 ± 13.2 | 56 | 68.8 ± 13.7 | 64 | 62.4 ± 12.5 | 26 | 80.8 ± 2.9 |
| DQ <75 | 56 | 60.0 ± 11.5 | 25 | 61.0 ± 12.1 | 31 | 59.2 ± 11.2 | 56 | 60.0 ± 11.5 | 0 | N/A |
| **PDMS-M DQ**^۵^ | 195 | 95.7 ± 18.1 | 61 | 90.0 ± 21.1 | 134 | 98.3 ± 16.0 | 72 | 82.6 ± 19.9 | 123 | 103.4 ± 11.4 |
| DQ <85 | 41 | 72 [59, 80] | 20 | 72.5 [56.5, 78] | 21 | 71 [65, 81] | 37 | 71 [59, 78] | 4 | 82 [81, 82.5] |
| DQ <75 | 23 | 61 [52, 70] | 12 | 59 [50.5, 71.5] | 11 | 65 [56, 70] | 23 | 61 [52, 70] | 0 | N/A |

Continuous data are expressed as mean ± standard deviation (SD) for normally distributed data and median [interquartile range, IQR] for non-normally distributed data

^۵^Corrected age (CA) was only used for infants ages 18 to <24 months

Abbreviations: AKI, acute kidney injury; NDI, neurodevelopmental impairment; CAT, clinical adaptive test; CLAMS, clinical linguistic and auditory milestone scale; PDMS-M,peabody developmental motor scale; DQ, developmental quotient; N/A, not applicable

Supplemental Table 3. Demographic Characteristics of Infants by Acute Kidney Injury (AKI) Status and Associated Outcomes

| **Demographic Characteristics** | **Overall**  **n=203** | **AKI** | | |
| --- | --- | --- | --- | --- |
|  |  | **Present**  n=64 (31.5) | **Absent**  n=139 (68.5) | ***p* value**^ح^ |
| Birth Weight (grams) | 1021 ± 277 | 829 ± 211 | 1110 ± 259 | **<0.001** |
| Gestational Age (weeks) | 27.9 ± 2.6 | 26.0 ± 2.1 | 28.8 ± 2.3 | **<0.001** |
| Male | 72 (35.5) | 31 (48.4) | 72 (51.8) | 0.656 |
| Race/Ethnicity |  |  |  | 0.109 |
| White/Caucasian | 74 (36.6) | 27 (42.2) | 47 (34.1) |  |
| Black/African American | 109 (54.0) | 30 (46.7) | 79 (57.3) |  |
| Hispanic | 14 (6.9) | 7 (10.9) | 7 (5.1) |  |
| Other | 5 (2.5) | 0 | 5 (3.6) |  |
| Reason for Delivery |  |  |  |  |
| Pre-eclampsia | 78 (38.4) | 17 (26.6) | 61 (43.9) | **0.018** |
| Chorioamnionitis | 11 (5.4) | 4 (6.3) | 7 (5.0) | 0.744 |
| Placenta Accreta | 0 | 0 | 0 | - |
| Placental Abruption | 20 (9.9) | 8 (12.5) | 12 (8.6) | 0.392 |
| Preterm Premature Rupture of Membranes | 30 (14.8) | 13 (20.3) | 17 (12.2) | 0.132 |
| IUGR | 17 (8.4) | 4 (6.3) | 13 (8.6) | 0.590 |
| Placental Insufficiency | 0 | 0 | 0 |  |
| Preterm Labor | 71 (35.0) | 28 (43.8) | 43 (30.9) | 0.0753 |
| Non-reassuring Fetal Heart Tones | 37 (18.2) | 10 (15.6) | 27 (19.4) | 0.515 |
| Abnormal Dopplers | 12 (5.9) | 5 (7.8) | 7 (5.0) | 0.524 |
| Maternal HELLP | 12 (5.9) | 5 (7.8) | 7 (5.0) | 0.524 |
| Other | 8 (3.9) | 2 (3.1) | 6 (4.3) | 1.000 |
| Size for Gestational Age |  |  |  | **0.007** |
| Small | 51 (25.1) | 8 (12.5) | 43 (30.9) |  |
| Average | 147 (72.4) | 53 (82.8) | 94 (67.6) |  |
| Large | 5 (2.5) | 3 (4.7) | 2 (1.4) |  |
| Apgar |  |  |  |  |
| 1 minute | 4.7 ± 2.3 | 4.2 ± 2.2 | 4.9 ± 2.2 | **0.0264** |
| 5 minutes | 7.1 ± 1.7 | 6.4 ± 2.0 | 7.5 ± 1.5 | **0.0002** |
| 10 minutes (n=61) | 7.1 ± 1.3 | 6.6 ± 1.5 | 7.4 ± 1.1 | **0.0123** |
| Abnormal Head Imaging during Admission^۵^ |  |  |  |  |
| No IVH | 131 (64.5) | 32 (50.0) | 99 (71.2) | **0.003** |
| Grade I/II | 53 (26.1) | 19 (29.7) | 34 (24.5) | 0.431 |
| Grade III/IV | 18 (8.9) | 12 (18.8) | 6 (4.3) | **<0.001** |
| PVL | 4 (2.0) | 2 (3.1) | 2 (1.4) | 0.592 |
| Supplemental FiO2 at Discharge | 43 (21.3) | 25 (39.7) | 18 (13.0) | **<.001** |
| AKI During Hospitalization (Discharge Diagnosis) | 40 (19.7) | 34 (53.1) | 6 (4.3) | **<.001** |
| Length of Hospitalization (days) | 79 [50.5, 110.5] | 110 [89, 127.5] | 64.5 [40, 89.5] | **<.001** |
| Developmental Clinic Follow Up Data |  |  |  |  |
| Adjusted Age at Visit (months) | 25.0 ± 4.4 | 25.2 ± 4.2 | 24.9 ± 4.6 | 0.662 |
| Chronologic Age at Visit (months) | 25.9 ± 3.5 | 26.0 ± 3.2 | 25.8 ± 3.6 | 0.763 |
| Weight at Visit (percentile) | 21 [5, 53] | 14 [3, 53] | 23.5 [9, 55] | **0.035** |
| Height at Visit (percentile) | 24 [9, 53] | 17 [5, 41] | 28.0 [11.5, 56] | **0.031** |
| Head Circumference at Visit (percentile) | 31 [11, 67] | 14.5 [4, 48] | 35 [15, 72] | **<0.001** |
| Gastrostomy Tube Present at Visit | 14 (6.9) | 10 (15.6) | 4 (2.9) | **0.002** |
| Support Services in use at Visit^۱^ |  |  |  |  |
| BabyNet (Early Intervention) | 128 (63.1) | 39 (60.9) | 89 (64.0) | 0.672 |
| Physical Therapy | 67 (33.0) | 30 (46.9) | 37 (26.6) | **0.004** |
| Occupational Therapy | 94 (46.3) | 36 (56.3) | 58 (41.7) | 0.054 |
| Speech Therapy | 98 (48.3) | 40 (62.5) | 58 (41.7) | **0.006** |
| None | 54 (26.6) | 12 (18.8) | 42 (30.2) | 0.086 |
| Insurance |  |  |  | 0.546 |
| Medicaid | 155 (76.4) | 47 (73.4) | 108 (77.7) |  |
| Private | 40 (19.7) | 13 (20.3) | 27 (19.4) |  |
| Tricare | 5 (2.5) | 3 (4.7) | 2 (1.4) |  |
| Self-Pay | 1 (0.5) | 0 | 1 (0.7) |  |
| None | 2 (0.5) | 1 (1.6) | 1 (0.7) |  |

Categorical data presented as count (proportion of column total). Continuous data presented as mean ± standard deviation or median [Q1, Q3];

^۵^Infants can have both IVH and PVL; ^۱^Multiple services may be used per infant

Abbreviations: IUGR, intrauterine growth restriction; HELLP, hemolysis, elevated liver enzymes, low platelets; IVH, intraventricular hemorrhage; PVL, periventricular

leukomalacia; Fi02, fractional inspired oxygen; AKI, acute kidney injury.

^ح^*p*-values Chi Square or Fisher’s Exact Test, Student’s Ttest or Wilcoxon Rank Sum Test
